# Strategies for Enhancing the Accumulation and Retention of Extracellular Matrix in Tissue-Engineered Cartilage Cultured in Bioreactors

**DOI:** 10.1371/journal.pone.0023119

**Published:** 2011-08-15

**Authors:** Kifah Shahin, Pauline M. Doran

**Affiliations:** 1 School of Biotechnology and Biomolecular Sciences, University of New South Wales, Sydney, New South Wales, Australia; 2 Department of Chemical Engineering, School of Biological Sciences, Monash University, Melbourne, Victoria, Australia; Pennington Biomedical Research Center, United States of America

## Abstract

Production of tissue-engineered cartilage involves the synthesis and accumulation of key constituents such as glycosaminoglycan (GAG) and collagen type II to form insoluble extracellular matrix (ECM). During cartilage culture, macromolecular components are released from nascent tissues into the medium, representing a significant waste of biosynthetic resources. This work was aimed at developing strategies for improving ECM retention in cartilage constructs and thus the quality of engineered tissues produced in bioreactors. Human chondrocytes seeded into polyglycolic acid (PGA) scaffolds were cultured in perfusion bioreactors for up to 5 weeks. Analysis of the size and integrity of proteoglycans in the constructs and medium showed that full-sized aggrecan was being stripped from the tissues without proteolytic degradation. Application of low (0.075 mL min^−1^) and gradually increasing (0.075–0.2 mL min^−1^) medium flow rates in the bioreactor resulted in the generation of larger constructs, a 4.0–4.4-fold increase in the percentage of GAG retained in the ECM, and a 4.8–5.2-fold increase in GAG concentration in the tissues compared with operation at 0.2 mL min^−1^. GAG retention was also improved by pre-culturing seeded scaffolds in flasks for 5 days prior to bioreactor culture. In contrast, GAG retention in PGA scaffolds infused with alginate hydrogel did not vary significantly with medium flow rate or pre-culture treatment. This work demonstrates that substantial improvements in cartilage quality can be achieved using scaffold and bioreactor culture strategies that specifically target and improve ECM retention.

## Introduction

Millions of people in all age groups suffer the debilitating effects of injury or disease of articular cartilage with incidence increasing in the elderly. Cartilage damage is commonly initiated by trauma, autoimmune disease, or osteoarthritis and may develop into a condition of irreversible deterioration. Tissue engineering of cartilage is a cell-based approach for the treatment of joints affected by irreparable cartilage damage [1], offering the potential for better clinical outcomes than can be achieved using current surgical practices and prostheses.

The quality of cartilage produced *in vitro* using tissue engineering techniques is determined by many parameters including cell source, cell expansion method, choice of scaffold for cell attachment, seeding technique, culture environment, nutrients, differentiation factors, and mechanical stimulation. Porous three-dimensional scaffolds are an integral component, distinguishing tissue engineering from standard cell culture techniques. The scaffold provides physical cues to the attached cells and can mimic extracellular matrix (ECM) in guiding cell differentiation while allowing nutrient and waste exchange with the environment. Poly(α-hydroxy ester)s such as polyglycolic acid (PGA), polylactic acid, and their co-polymers are of particular interest as scaffold materials because they are biodegradable, approved for surgical use, and widely used clinically in humans.

Culture of seeded scaffolds in a dynamic environment involving fluid flow or mixing is beneficial for cartilage synthesis compared with static culture conditions [2]–[5]. Various bioreactor devices have been applied for cartilage tissue engineering [6], [7], offering advantages such as better control over culture conditions, reduced diffusional limitations for delivery of nutrients and metabolites, enhanced oxygen transfer and gas exchange, and exertion of mechanical and hydrodynamic forces influencing cell and tissue development. Bioreactor cultivation periods used for cartilage production range from days to months. Direct perfusion or recirculation bioreactors, which have a relatively simple configuration and are designed to force a recirculating flow of culture medium through porous cell-seeded scaffolds, have been shown in several studies to improve cartilage ECM production compared with static culture systems [8]–[10].

Theoretical studies have been used to calculate the medium flow rates required in bioreactors to deliver adequate oxygen and nutrients in cartilage cultures [11], [12] and to exert flow-induced shear stresses suitable for mechanical signal transduction in the cells [13]. Yet, flow of medium through nascent constructs has the potential to strip ECM components such as glycosaminoglycan (GAG) and collagen from the tissues, thus hindering cartilage formation. Loss of ECM into the medium after synthesis represents a substantial waste of resources and cellular activity in cartilage cultures. The quantity of material released reflects to some extent the porosity and structural properties of the scaffold and developing matrix but is also affected by the hydrodynamic and other operating conditions applied during bioreactor culture [3], [10], [14].

Typically, the concentration of collagen achieved in tissue-engineered cartilage is substantially lower than that in native articular cartilage [2], [5], [15]–[17]. Because networks of collagen type II fibrils are responsible for the tensile strength of cartilage, tissue-engineered constructs generally exhibit inferior mechanical properties compared with native articular cartilage [18], [19]. Collagen networks also play an important role in the retention of macromolecules within developing tissues: for example, collagen is necessary for the retention of newly synthesized proteoglycans to form insoluble cartilage matrix [20]. Collagen in tissue-engineered cartilage may not be fully assembled into thick collagen fibrils [21], [22]: as in fetal cartilage, it is likely that the C- and/or N- terminal propeptides of extracellular procollagen molecules remain in place, thus preventing final aggregation into the banded fibrils characteristic of mature cartilage [23], [24]. Under these conditions, GAG retention in the tissues may be compromised by the relatively loose structure of the prevailing procollagen network. On the other hand, in the same way that proteolysis and turnover of proteoglycans occur routinely in cartilage *in vivo* [25], it is also possible that GAG is lost from cartilage constructs as a consequence of proteoglycan degradation and the formation of fragments that are easily removed from the tissues, especially under flow conditions.

In previous work in this laboratory, recirculation perfusion bioreactors were developed for cartilage tissue engineering using PGA scaffolds [16]. However, loss of up to 72% of ECM components from the constructs into the medium was identified as a significant problem reducing the overall quality of the tissues generated. The aim of the current work was to establish new culture protocols to address this issue and minimize ECM losses during bioreactor operation. Several approaches were taken. PGA–alginate scaffolds were tested to determine whether the presence of hydrogel within the interstices of fibrous PGA mesh could improve ECM retention compared with PGA alone. Different bioreactor operating conditions including high, low, and gradually increasing flow rate regimes were evaluated for their effect on ECM loss and cartilage composition. In addition, as a protective strategy to improve subsequent tissue retention, scaffold pre-culture was used prior to perfusion culture to allow some cartilage matrix to be deposited within the scaffolds before imposition of medium flow. New information about the mechanism of GAG loss from the constructs was obtained by analyzing the integrity of proteoglycan complexes recovered from the culture medium to identify whether proteolytic processing affected GAG retention. Human chondrocytes were employed in this work as a more pertinent system for clinical applications than animal models. Human fetal cartilage cells, although not fully differentiated into mature chondrocytes, have been shown previously to possess a greater capacity for cartilage synthesis than human mesenchymal stem cells [26] and have the advantage of faster growth rate, greater developmental plasticity, and thus easier manipulation compared with adult chondrocytes.

## Materials and Methods

### Cells, scaffolds and seeding

This research was conducted with approval from the University of New South Wales Human Research Ethics Committee. Chondrocytes were isolated from human fetal epiphyseal cartilage in knee and hip joints obtained with written informed parental consent after 16–20 weeks of gestation. The cells were expanded over two passages (P2) in monolayer as described previously [17]. The scaffolds were disks of fibrous PGA mesh of bulk density 58 mg cm^−3^, porosity 94%, and fiber diameter 12–15 µm (Albany International Research, Mansfield, USA). The disk diameter was 15 mm and the disk thickness was 4.6 mm.

The scaffolds were seeded using semi-static and PGA–alginate loading methods [17] and 20×10^6^ P2 cells. Briefly, for semi-static seeding, suspended cells were loaded into PGA disks in well plates using a pipette, the scaffolds were turned over manually for the first 2.5 h to encourage uniform distribution of cells within the disks, and incubation was carried out for 3 days in shaking T-flasks positioned at an angle of about 30° above horizontal on a rotary shaker operated at 65 rpm. For PGA–alginate seeding, cells suspended in a solution containing 1.2% sodium alginate were loaded into PGA disks using a pipette. The scaffolds were treated with CaCl_2_ to gelify the alginate, transferred to shaking T-flasks, and incubated for 3 days as described for semi-static seeding. After seeding, there was no significant difference in cell density between the PGA and PGA–alginate scaffolds [17].

### Bioreactor cultures

Seeded scaffolds were cultured at 37°C in triplicate custom-built recirculation column bioreactors [16] in a 5% CO_2_ incubator. Each bioreactor was operated using 200 mL of complete medium [17]. The culture conditions tested are summarized in Table 1. Scaffolds in the bioreactors were perfused with medium using three different flow rate regimes. Constant volumetric flow rates of 0.2 mL min^−1^ (high flow rate) and 0.075 mL min^−1^ (low flow rate) were used, corresponding to superficial linear velocities ( = volumetric flow rate/reactor cross-sectional area) of 19 µm s^−1^ and 7 µm s^−1^, respectively. In other experiments, a gradual increase in flow rate was applied, starting from 0.075 mL min^−1^ at the beginning of the culture and increasing by about 0.025 mL min^−1^ each week and twice in the last week to give a final flow rate of 0.2 mL min^−1^. Some seeded scaffolds were pre-cultured in 150-cm^2^ shaking T-flasks containing 200 mL of complete medium and one construct per flask for either 5 days or 2.5 weeks prior to bioreactor culture at a constant medium flow rate of 0.2 mL min^−1^ (Table 1). Non-perfused control cultures were also carried out for 5 weeks in shaking T-flasks. During all bioreactor and T-flask cultures and pre-cultures, 100 mL of spent medium was removed and replaced with fresh medium every 3 days or twice per week. In the bioreactors, the flow direction was reversed each time the medium was exchanged: irrespective of the direction of liquid flow through the scaffolds, medium always flowed against gravity as the bioreactor chambers were inverted after each change in flow direction. All bioreactor and T-flask experiments were conducted in triplicate.

**Table 1:** Scaffolds and culture conditions tested.

Experiment	Scaffold	T-flask culture period	Bioreactor culture period	Bioreactor medium flow rate (mL min^−1^)
1	PGA	5 weeks	NA	NA
2	PGA	0	5 weeks	0.2 (high)
3	PGA	0	5 weeks	0.075 (low)
4	PGA	0	5 weeks	0.075–0.2 (gradually increasing)
5	PGA	5 days	30 days	0.2 (high)
6	PGA	2.5 weeks	2.5 weeks	0.2 (high)
7	PGA–alginate	0	5 weeks	0.2 (high)
8	PGA–alginate	0	5 weeks	0.075–0.2 (gradually increasing)
9	PGA–alginate	5 days	30 days	0.2 (high)

NA = not applicable.

All cultures were conducted using triplicate T-flasks and/or bioreactors.

Cartilage constructs were harvested after a total cultivation time after seeding of 5 weeks. The harvested scaffolds were washed, weighed, and sectioned for biochemical and histological assays as described previously [17]. Samples of spent culture medium were stored at −20°C for analysis.

### Biochemical and histological analyses

Tissue-engineered cartilage was analyzed for wet weight, dry weight, water content, and cell, GAG, total collagen, and collagen type II concentrations as described previously [17]. GAG and hydroxyproline concentrations were also measured in medium samples. For correct estimation of GAG release into the medium, GAG was determined to be stable in the medium for at least 2 weeks under the culture conditions employed (37°C and 5% CO_2_). Alginate residue in cartilage tissues produced using PGA–alginate scaffolds was measured by weighing small tissue sections before and after incubation in alginate-dissolving buffer containing 0.15 M NaCl and 55 mM tri-sodium citrate with gentle shaking for 10 min at 37°C.

Samples were prepared for histological analysis as described previously [17]. Tissue sections were immunostained with monoclonal antibodies against collagen type I (clone I-8H5: ICN Biomedicals, Seven Hills, Australia) and collagen type II (clone II-4C11: ICN Biomedicals) using a Bond automated immunostainer and Bond Polymer Refine Detection Kit (Leica Microsystems, North Ryde, Australia). The kit contained hydrogen peroxide to block endogenous peroxidase, serum for protein blocking, secondary immunoglobulins, polymer tertiary solution, diaminobenzidine chromogen, and haematoxylin counterstain. The primary antibodies were used at dilutions of 1∶20,000 for collagen type I and 1∶2000 for collagen type II and the incubation time was 30 min. Before immunostaining, the hydrated tissue sections were treated for 30 min at 37°C with 0.2% hyaluronidase (testicular type III: Sigma, St Louis, USA) in Tris-buffered saline (TBS: prepared as a 10× solution using 1 M Tris base, 2 M NaCl, and 50 mM CaCl_2_ in Milli-Q water, pH 8.0) to remove proteoglycans, and then treated with an antigen retrieval solution (Novacastral™: Leica Microsystems) for 20 min at 95°C, washing with TBS after each step.

### Proteoglycan extraction and analysis

Proteoglycans were isolated from tissue-engineered constructs and human fetal cartilage using the guanidine extraction method [27]. The extract was cleared by centrifugation, dialyzed for 48 h using multiple changes of distilled water at 4°C, and then freeze-dried. To prevent interaction between highly charged proteoglycans and other molecules in the spent medium, guanidine-HCl was added at a concentration of 4 M [28]. The medium was dialyzed against water and then freeze-dried.

A composite gel containing 1.2% acrylamide and 0.6% agarose was prepared for electrophoresis of intact proteoglycans [29]–[31]. The gel was cast into 8 cm×8 cm×1.5 mm slabs. Freeze-dried samples were reconstituted in sample buffer [31] to give a concentration of 0.25 µg µL^−1^ GAG, heated for 5 min in boiling water, and loaded on to the gel using a volume of 10 µL. Two gels containing the same samples were run in parallel. Using 0.04 M cold Tris-acetate as electrode buffer, the gels were pre-run for 1 h at 120 V to remove unpolymerized acrylamide [31]. The electrode buffer was then changed and the samples were loaded and separated in the cold for 2 h using a current of 28 mA per gel. The potential difference between the upper and lower buffer compartments was around 90 V. Bovine aggrecan (Sigma) and shark chondroitin sulphate (Sigma) were used as size markers. To evaluate the effectiveness of separation, bovine aggrecan was mixed 1∶1 w/w with chondroitin sulphate or 1∶1 w/w with proteoglycan isolated from fetal cartilage. Proteoglycans in the culture medium formed aggregates and did not separate well on the gel. To dissociate the aggregates, the sample concentration was reduced to 0.08 µg µL^−1^ GAG by diluting with freshly made 6 M urea in 0.04 M Tris-acetate. Alternatively, samples were treated with 0.2% hyaluronidase in 0.04 M Tris-acetate for 30 min at 37°C before boiling and loading on to the gel.

After electrophoresis, the gels were fixed for 60 min using 50% methanol and 10% acetic acid in Milli-Q water, stained for at least 2 h with a solution of 0.025% toluidine blue in 3% acetic acid, destained for 2–3 h using 3% acetic acid in multiple changes, and then cleared overnight in water. Transblotting on nitrocellulose (pore size 0.45 µm: Invitrogen, Carlsbad, CA, USA) was carried out in the cold for 1.5 h at 100 V using a transfer buffer containing 5% methanol [31]. Two nitrocellulose membranes were placed one on top of the other against the gel to avoid the loss of small-size molecules during the transfer; the second membrane acted as a back-up to bind any proteins that might pass through the first membrane. The blotted membranes were immunodetected using monoclonal antibody against human aggrecan targeted specifically to the hyaluronic-acid-binding region of the aggrecan molecule (clone 969D4D11: Invitrogen). A WesternBreeze™ Chemiluminescent Detection kit (anti-mouse: Invitrogen) was used according to the manufacturer's instructions to detect bound primary antibodies. Visible chemiluminescence was imprinted and then developed on X-ray film.

### Statistical analysis

All culture experiments were performed in triplicate. Data are presented as averages ± standard errors. The Student's *t*-test was used to compare two groups of data; one-way analysis of variance (ANOVA) in conjunction with Fisher's Protected Least Significant Difference (PLSD) and Tukey–Kramer multiple-comparisons tests were used to compare three or more groups of data. When both multiple comparisons tests were in agreement at the 5% or less level, the *p* value of the Fisher's PLSD test is reported.

## Results

### Effect of bioreactor hydrodynamics and scaffold pre-culture using PGA scaffolds

Experiments were conducted with the aim of limiting the loss of ECM components from constructs into the medium during bioreactor culture. Two approaches were tested using PGA scaffolds. Low (0.075 mL min^−1^) and gradually increasing (0.075–0.2 mL min^−1^) medium flow rates were applied to determine if moderation of the hydrodynamic forces within the perfused scaffolds improved GAG retention compared with operation at 0.2 mL min^−1^ as used previously [16]. Pre-culture of scaffolds for 5 days or 2.5 weeks in T-flasks without perfusion was also examined to assess whether deposition of cartilage matrix prior to bioreactor culture could play a protective role in minimizing subsequent ECM losses under perfusion conditions.

**Figure 1:**
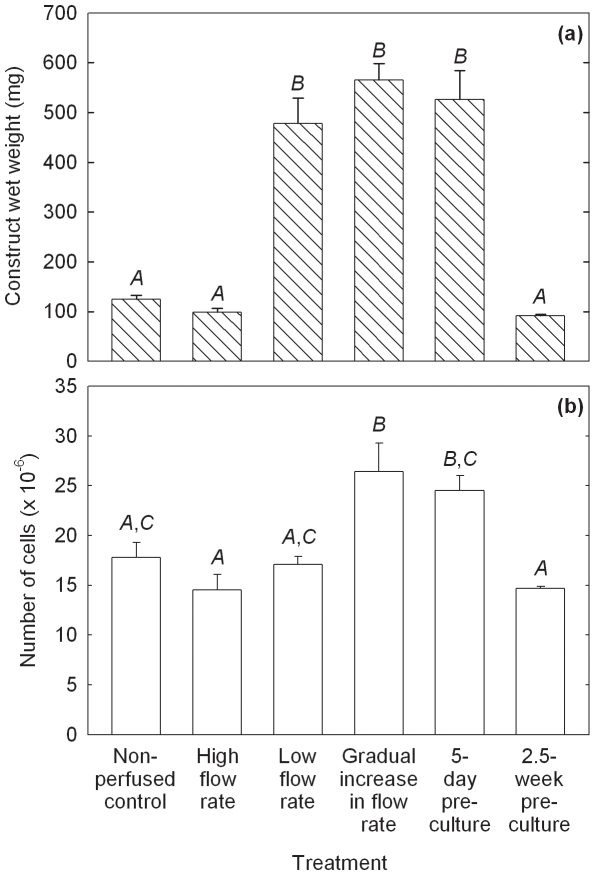
Properties of cartilage constructs produced using PGA scaffolds cultured in shaking T-flasks (non-perfused control), in perfusion bioreactors using a constant flow rate of 0.2 mL min^−1^ (high flow rate), a constant flow rate of 0.075 mL min^−1^ (low flow rate), or a gradually increasing flow rate of 0.075–0.2 mL min^−1^ (gradual increase in flow rate), or using scaffold pre-culture in T-flasks for either 5 days (5-day pre-culture) or 2.5 weeks (2.5-week pre-culture) prior to bioreactor culture at a constant flow rate of 0.2 mL min^−1^. (a) Construct wet weight; and (b) number of cells. The scaffolds were seeded using 20×10^6^ cells and cultured for a total of 5 weeks after seeding. The error bars represent standard errors from triplicate T-flask and/or bioreactor cultures. For each construct property, results labeled with different letters (*A*, *B*, *C*) are statistically different from each other (*p*<0.01).

**Figure 2:**
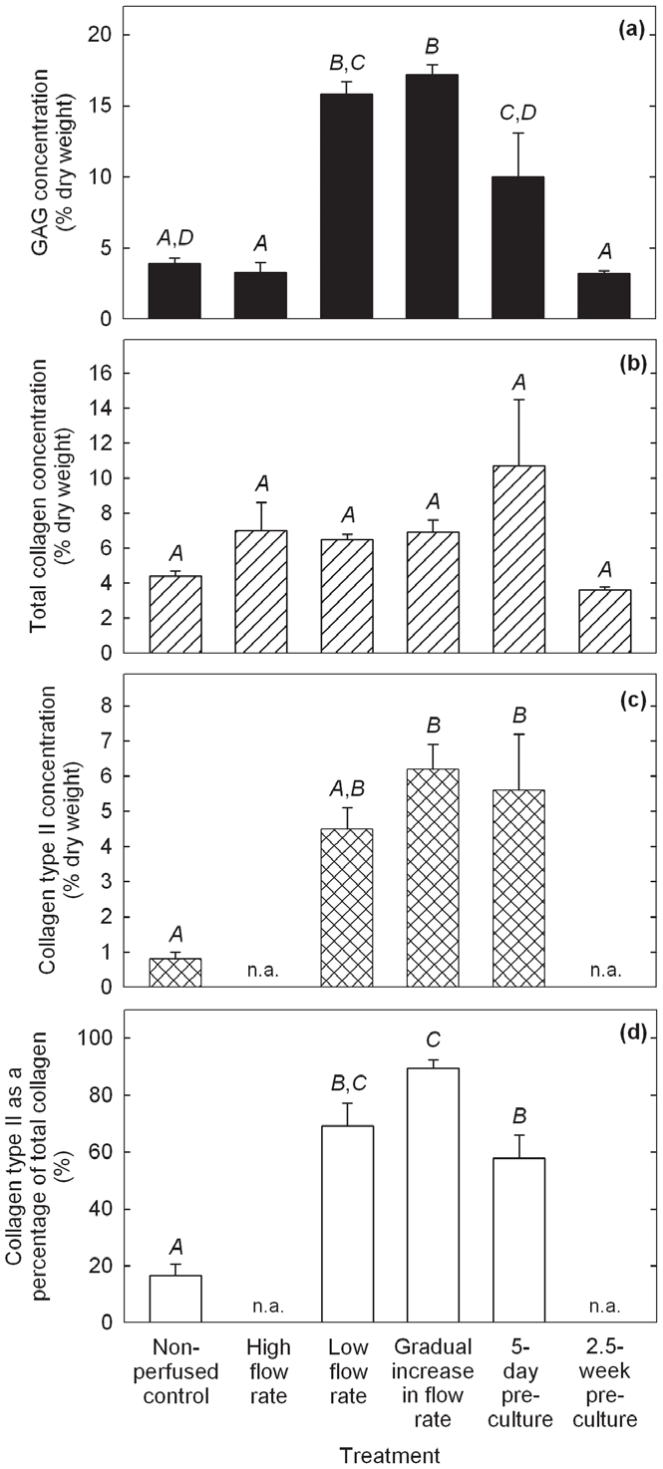
Biochemical properties of cartilage constructs produced using PGA scaffolds cultured in shaking T-flasks (non-perfused control), in perfusion bioreactors using a constant flow rate of 0.2 mL min^−1^ (high flow rate), a constant flow rate of 0.075 mL min^−1^ (low flow rate), or a gradually increasing flow rate of 0.075–0.2 mL min^−1^ (gradual increase in flow rate), or using scaffold pre-culture in T-flasks for either 5 days (5-day pre-culture) or 2.5 weeks (2.5-week pre-culture) prior to bioreactor culture at a constant flow rate of 0.2 mL min^−1^. (a) GAG concentration; (b) total collagen concentration; (c) collagen type II concentration; and (d) collagen type II as a percentage of total collagen. The scaffolds were seeded using 20×10^6^ cells and cultured for a total of 5 weeks after seeding. The error bars represent standard errors from triplicate T-flask and/or bioreactor cultures. n.a. = not analyzed. For each construct property, results labeled with different letters (*A*, *B*, *C*, *D*) are statistically different from each other (*p*<0.01).

Results from Experiments 1–6 (Table 1) for PGA scaffolds are shown in Figures 1 and 2. Relatively low construct wet weights were obtained for the non-perfused, high flow rate, and 2.5-week pre-culture groups (Fig. 1a). The construct wet weights formed using low and gradually increasing flow rates were 4.8-fold (*p*<0.0001) and 5.7-fold (*p*<0.0001) higher, respectively, than those in the high flow rate cultures. Pre-culture of scaffolds for 5 days prior to bioreactor culture also improved the construct wet weight 5.3-fold (*p*<0.0001) relative to high flow rate operation. These results are consistent with visual observations of construct shrinkage in the non-perfused, high flow rate, and 2.5-week pre-culture experiments after Days 20–25 of the 5-week culture period: construct shrinkage did not occur when low and gradually increasing medium flow rates were used or when the scaffolds were pre-cultured for 5 days. The water contents of the constructs were (86.4±0.7)%, (86.3±1.6)%, (91.4±0.0)%, (89.6±0.3)%, (88.7±1.2)%, and (85.9±0.6)% for the non-perfused control, high flow rate, low flow rate, gradual increase in flow rate, 5-day pre-culture, and 2.5-week pre-culture groups, respectively. The numbers of cells in constructs harvested from the high flow rate, low flow rate, and 2.5-week pre-culture experiments were not significantly different from those in the non-perfused controls (Fig. 1b). In contrast, cell numbers after applying a gradual increase in medium flow rate or a 5-day pre-culture period were 1.8-fold (*p* = 0.0003) and 1.7-fold (*p* = 0.0010) higher, respectively, than in the high flow rate cultures.

Constructs from the low and gradually increasing flow rate experiments contained 4.8-fold (*p*<0.0001) and 5.2-fold (*p*<0.0001) higher GAG concentrations, respectively, compared with those in the high flow rate cultures (Fig. 2a). GAG concentrations in the 5-day pre-culture experiment were 3.0-fold greater (*p* = 0.005) than in the high flow rate cultures; however, GAG concentrations in the 2.5-week pre-culture group were not different statistically from those in the non-perfused and high flow rate experiments. There was no significant difference in total collagen concentration between any of the treatment groups (Fig. 2b). Collagen type II concentrations were not significantly different in constructs produced using the low flow rate, gradually increasing flow rate, and 5-day pre-culture treatments: collagen type II was not measured in the high flow rate and 2.5-week pre-culture constructs (Fig. 2c). Collagen type II concentrations using the gradually increasing flow rate and 5-day pre-culture treatments were improved 7.8-fold (*p* = 0.0032) and 7.0-fold (*p* = 0.0064), respectively, compared with the non-perfused controls. Results for collagen type II as a percentage of total collagen were also significantly higher using the low flow rate (*p* = 0.0003), gradually increasing flow rate (*p*<0.0001), and 5-day pre-culture (*p* = 0.0016) regimes relative to the non-perfused controls (Fig. 2d).

**Figure 3:**
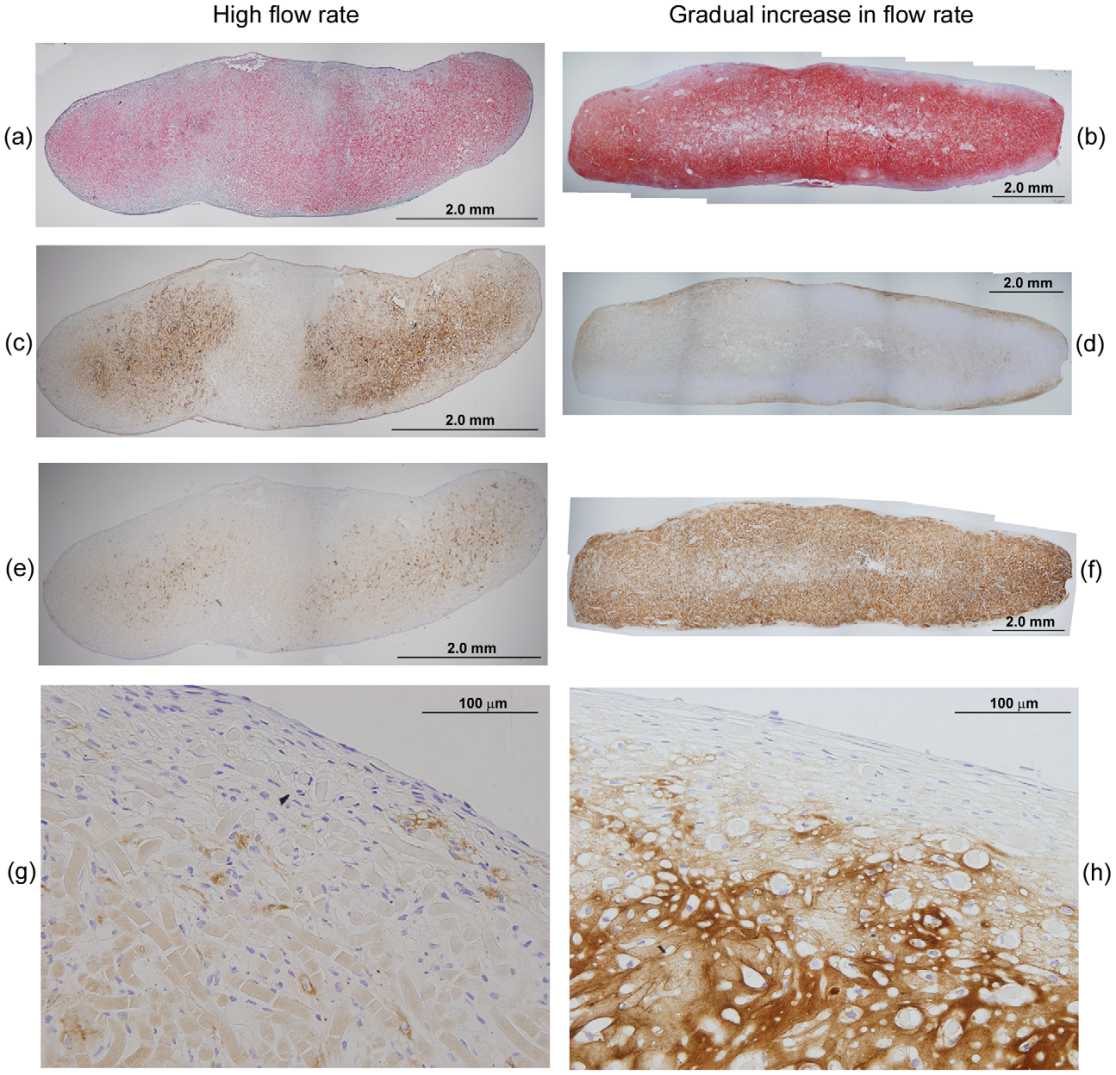
Histological appearance of constructs produced using PGA scaffolds cultured in bioreactors for 5 weeks at a constant flow rate of 0.2 mL min^−1^ (high flow rate: a, c, e, g) or gradually increasing flow rate of 0.075–0.2 mL min^−1^ (gradual increase in flow rate: b, d, f, h). The scaffolds were seeded using 20×10^6^ cells. Construct cross-sections show: (a, b) pink–red staining for GAG; (c, d) immunostaining (brown) for collagen type I; (e, f) immunostaining (brown) for collagen type II; and (g, h) immunostaining (brown) for collagen type II and blue–purple staining for cells at high magnification.

The histological appearance of constructs from the high flow rate and gradually increasing flow rate cultures is shown in Figure 3. The smaller size of the tissues produced under high flow rate conditions (length 6.9 mm, maximum thickess 2.3 mm) compared with those produced with gradually increasing flow rate (length 14 mm, maximum thickess 3.5 mm) (Figs. 3a, 3b) is consistent with visual observations of construct shrinkage in the high flow rate experiments. Operating the bioreactor with gradually increasing flow rate produced tissues with more intense staining for GAG compared with those produced at high flow rate (Figs. 3a, 3b), reflecting the quantitative results for GAG concentration (Fig. 2a). Both cultures generated contructs that stained positively for collagen type I (Figs. 3c, 3d); collagen type I was localized mainly in the peripheral regions after the gradual increase in flow rate treatment compared with a mostly internal distribution in the high flow rate constructs. The development of low GAG/high collagen type I capsules at the periphery of cartilage constructs has been observed previously [15], [16] and has been attributed to high fluid shear, mixing, and turbulence at the construct surface [2]. Immunostaining for collagen type II was more intense and uniformly distributed in constructs produced using a gradual increase in flow rate compared with high flow rate conditions (Figs. 3e, 3f). Higher magnification of sections immunostained for collagen type II (Figs. 3g, 3h) showed fewer undissolved PGA fibers and cells of more rounded shape surrounded by pericellular regions in constructs from the gradual increase in flow rate cultures compared with those at high flow rate. The presence of undissolved PGA fibers in the high flow rate construct (seen in the lower half of Fig. 3g) is consistent with tissue shrinkage and medium by-passing during the later stages of the culture period.

**Figure 4:**
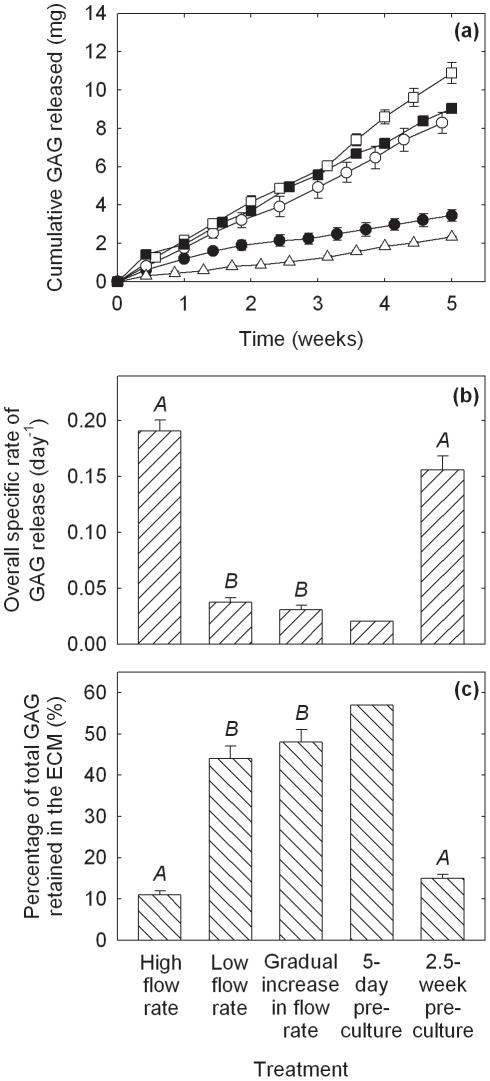
GAG release into the medium and retention in the constructs for PGA scaffolds cultured in bioreactors operated using a constant flow rate of 0.2 mL min^−1^ (high flow rate, •), a constant flow rate of 0.075 mL min^−1^ (low flow rate, ○), a gradually increasing flow rate of 0.075–0.2 mL min^−1^ (gradual increase in flow rate, □), or scaffold pre-culture in T-flasks for either 5 days (5-day pre-culture, ▪) or 2.5 weeks (2.5-week pre-culture, ▵) prior to bioreactor culture at a constant flow rate of 0.2 mL min^−1^. (a) Cumulative amount of GAG released into the medium; (b) overall specific rate of GAG release (mg per day per mg of GAG in the constructs at harvest); and (c) percentage of total GAG (construct+medium) retained in the constructs. The scaffolds were seeded using 20×10^6^ cells and cultured for a total of 5 weeks after seeding. The error bars represent standard errors from triplicate bioreactor cultures. Medium GAG data for the 5-day pre-culture treatment were measured in only one of the triplicate bioreactors and are thus unreplicated. In (b) and (c), results labeled with different letters (*A*, *B*) are statistically different from each other (*p*<0.0001).

Results for GAG release into the medium and retention in the PGA constructs are shown in Figure 4. The cumulative amounts of GAG released into the medium were higher for the low flow rate, gradually increasing flow rate, and 5-day pre-culture treatments compared with the high flow rate and 2.5-week pre-culture groups (Fig. 4a). The average rates of GAG release into the medium were 0.082, 0.24, 0.31, 0.25, and 0.064 mg day^−1^ for the high flow rate, low flow rate, gradually increasing flow rate, 5-day pre-culture, and 2.5-week pre-culture groups, respectively. Thus, the rate of GAG release correlated roughly with the concentration of GAG in the tissues (Figs. 4a, 2a). As the rate of GAG release can be expected to increase with increasing GAG concentration in the constructs irrespective of the medium flow or pre-culture conditions, calculation of the overall specific rate of GAG release into the medium (mg per day per mg of GAG in the constructs at harvest) provides a more useful indicator of relative GAG retention between the treatment groups. Results for the overall specific rate of GAG release (Fig. 4b) highlight the superior relative levels of GAG retention associated with the low flow rate, gradually increasing flow rate, and 5-day pre-culture treatments. The total amount of GAG (construct+medium) in the cultures at harvest was determined and the percentage of total GAG retained within the ECM calculated. The percentage of total GAG retained in the tissues for the low and gradually increasing flow rate treatments was 2.9–4.4-fold greater (*p*<0.0001) than in the high flow rate and 2.5-week pre-culture groups (Fig. 4c). An unreplicated measurement of medium GAG from the 5-day pre-culture experiment yielded a 3.8–5.2-fold increase in percentage GAG retention relative to the high flow rate and 2.5-week pre-culture groups but was not included in the statistical analysis. In summary, although greater amounts of GAG were lost to the medium during the low flow rate, gradually increasing flow rate, and 5-day pre-culture experiments (Fig. 4a), total GAG accumulation was also improved in these cultures so that the proportion of total GAG lost into the medium was only 43–56% compared with 89% using high flow rate operation (Fig. 4c).

### Effect of PGA–alginate scaffolds

The bioreactors in experiments with PGA–alginate scaffolds were operated using a constant high flow rate of 0.2 mL min^−1^ or a gradually increasing flow rate of 0.075–0.2 mL min^−1^. The effect of pre-culturing the scaffolds in T-flasks for 5 days prior to bioreactor culture at 0.2 mL min^−1^ was also tested. No shrinkage of the PGA–alginate constructs was observed during these experiments and there was negligible alginate residue (ca. 0.1 µg) in the tissues at harvest.

**Figure 5:**
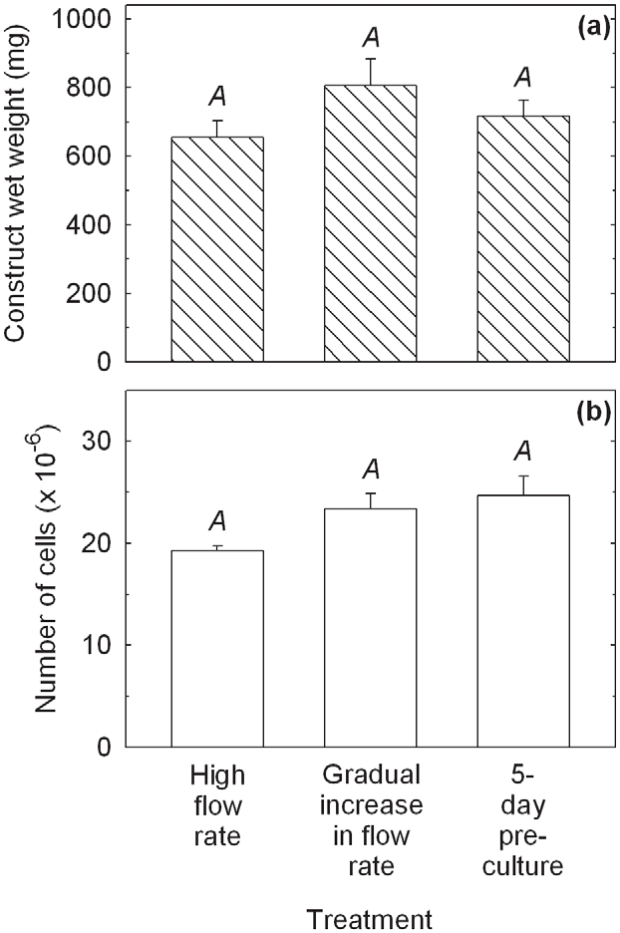
Properties of cartilage constructs produced using PGA–alginate scaffolds cultured in bioreactors using a constant flow rate of 0.2 mL min^−1^ (high flow rate), a gradually increasing flow rate of 0.075–0.2 mL min^−1^ (gradual increase in flow rate), or scaffold pre-culture in T-flasks for 5 days prior to bioreactor culture at a constant flow rate of 0.2 mL min^−1^ (5-day pre-culture). (a) Construct wet weight; and (b) number of cells. The scaffolds were seeded using 20×10^6^ cells and cultured for a total of 5 weeks after seeding. The error bars represent standard errors from triplicate bioreactors.

**Figure 6:**
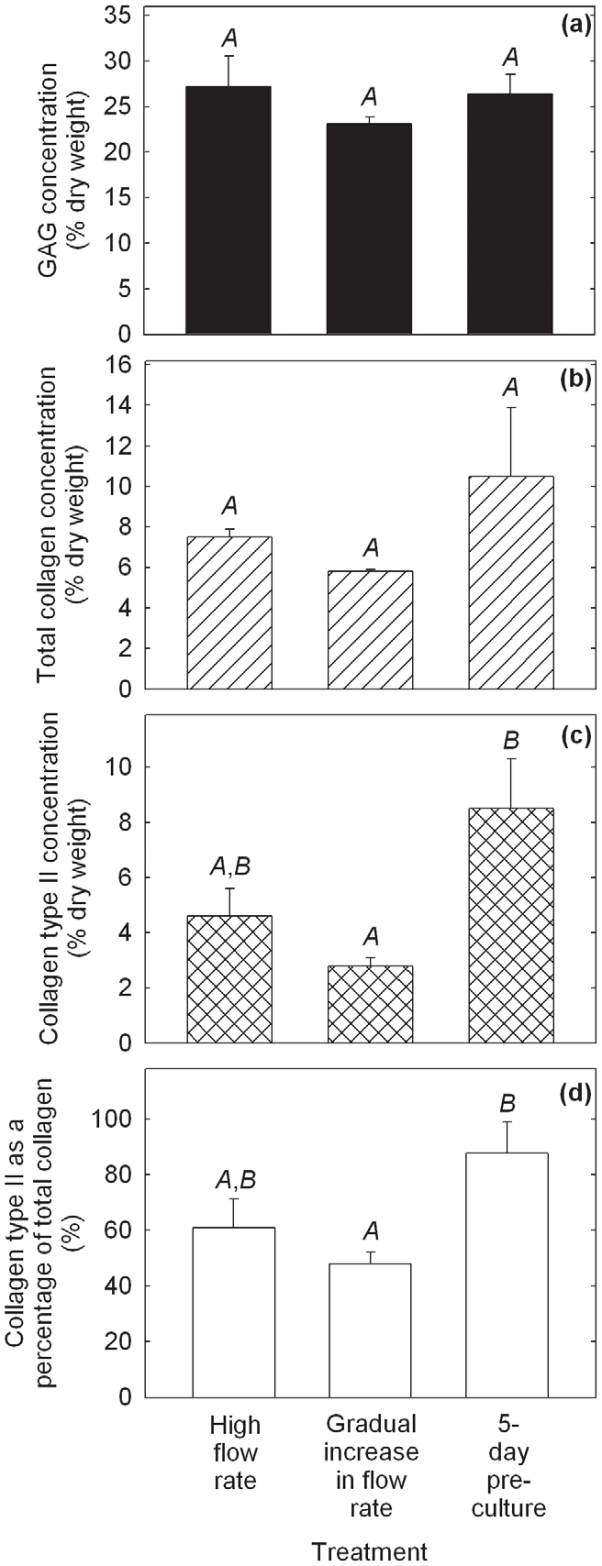
Biochemical properties of cartilage constructs produced using PGA–alginate scaffolds cultured in bioreactors using a constant flow rate of 0.2 mL min^−1^ (high flow rate), a gradually increasing flow rate of 0.075–0.2 mL min^−1^ (gradual increase in flow rate), or scaffold pre-culture in T-flasks for 5 days prior to bioreactor culture at a constant flow rate of 0.2 mL min^−1^ (5-day pre-culture). (a) GAG concentration; (b) total collagen concentration; (c) collagen type II concentration; and (d) collagen type II as a percentage of total collagen. The scaffolds were seeded using 20×10^6^ cells and cultured for a total of 5 weeks after seeding. The error bars represent standard errors from triplicate bioreactors. Results labeled with different letters (*A*, *B*) are statistically different from each other (*p*<0.05).

Results from analysis of constructs produced using PGA–alginate scaffolds in Experiments 7–9 (Table 1) are shown in Figures 5 and 6. There was no significant difference in tissue wet weight, number of cells, GAG concentration, or total collagen concentration between any of the groups tested (Figs. 5a, 5b, 6a, 6b). The water contents of the constructs were (94.5±0.4)%, (92.0±0.3)%, and (89.8±0.3)% for the high flow rate, gradual increase in flow rate, and 5-day pre-culture groups, respectively. Collagen type II concentrations and levels of collagen type II as a percentage of total collagen were 3.0-fold (*p* = 0.0159) and 1.8-fold (*p* = 0.0208) greater, respectively, after the 5-day pre-culture treatment than for the gradual increase in flow rate group (Figs. 6c, 6d).

**Figure 7:**
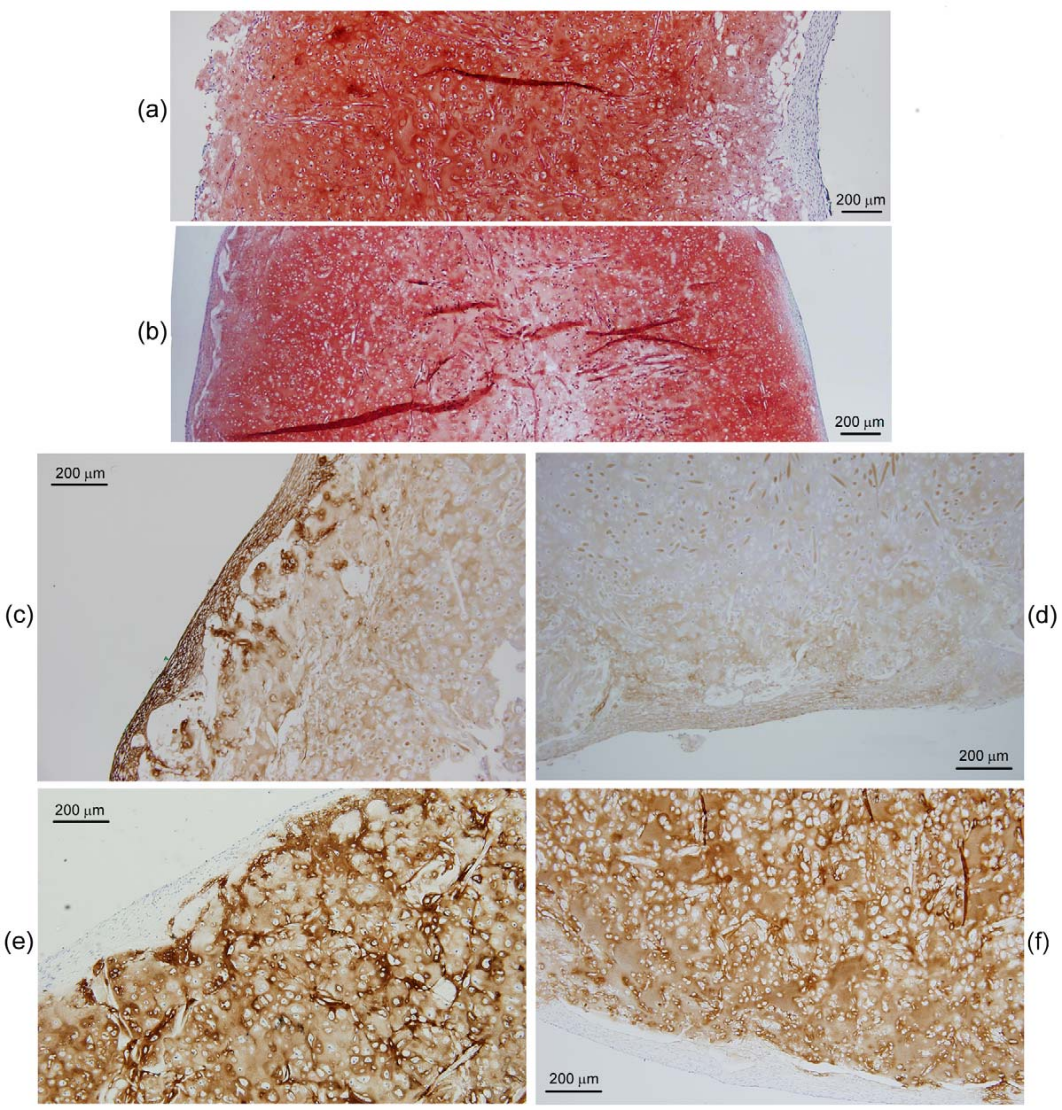
Histological appearance of constructs produced using PGA–alginate scaffolds cultured in bioreactors for 5 weeks at a constant high flow rate of 0.2 mL min^−1^ (a, c, e) or a gradually increasing flow rate of 0.075–0.2 mL min^−1^ (b, d, f). The scaffolds were seeded using 20×10^6^ cells. Construct cross-sections show: (a, b) pink–red staining for GAG, blue staining for collagen, and dark blue–purple staining for cells; (c, d) immunostaining (brown) for collagen type I; and (e, f) immunostaining (brown) for collagen type II.

The histological appearance of PGA–alginate constructs from the high flow rate and gradually increasing flow rate experiments is shown in Figure 7. The staining intensity for GAG was similar in the two cultures (Figs. 7a, 7b) consistent with the quantitative results for GAG concentration (Fig. 6a). Operating the bioreactor at high flow rate produced tissues with a more pronounced peripheral capsule of collagen type I compared with constructs from the gradually increasing flow rate cultures (Figs. 7c, 7d). The staining intensity for collagen type II was similar in the two cultures (Figs. 7e, 7f) consistent with the quantitative results for collagen type II concentration (Fig. 6c).

**Figure 8:**
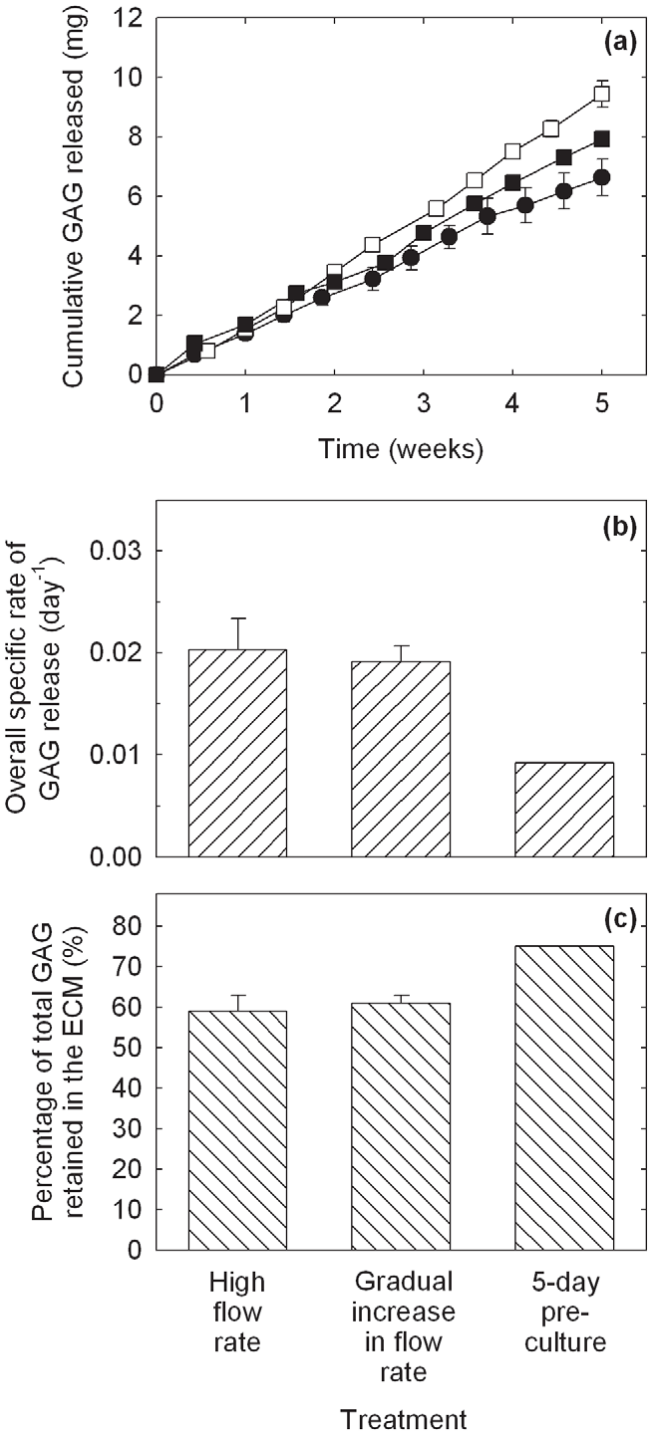
GAG release into the medium and retention in the constructs for PGA–alginate scaffolds cultured in bioreactors operated using a constant flow rate of 0.2 mL min^−1^ (high flow rate, •), a gradually increasing flow rate of 0.075–0.2 mL min^−1^ (gradual increase in flow rate, □), or scaffold pre-culture in T-flasks for 5 days prior to bioreactor culture at a constant flow rate of 0.2 mL min^−1^ (5-day pre-culture, ▪). (a) Cumulative amount of GAG released into the medium; (b) overall specific rate of GAG release (mg per day per mg of GAG in the constructs at harvest); and (c) percentage of total GAG (construct+medium) retained in the constructs. The scaffolds were seeded using 20×10^6^ cells and cultured for a total of 5 weeks after seeding. The error bars represent standard errors from triplicate bioreactors. Medium GAG data for the 5-day pre-culture treatment were measured in only one of the triplicate bioreactors and are thus unreplicated.

Results for GAG release into the medium and retention in the constructs for the PGA–alginate scaffolds are shown in Figure 8. Similar amounts of GAG were released during the three treatments for most of the culture period (Fig. 8a); the average rates of GAG release into the medium were 0.19, 0.28, and 0.22 mg day^−1^ for the high flow rate, gradually increasing flow rate, and 5-day pre-culture treatments, respectively. There was no significant difference in the overall specific rate of GAG release into the medium (mg per day per mg of GAG in the constructs at harvest) between the high and gradually increasing flow rate treatments (Fig. 8b); the result using an unreplicated measurement of medium GAG from the 5-day pre-culture treatment not included in the statistical analysis is also shown in Figure 8b. Overall specific rates of GAG release from the PGA–alginate scaffolds (Fig. 8b) were roughly similar to those obtained for the low flow rate, gradually increasing flow rate, and 5-day pre-culture treatments using PGA scaffolds without alginate (Fig. 4b). In contrast, results for the high flow rate cultures using PGA–alginate (Fig. 8b) were about an order of magnitude lower than for the high flow rate cultures without alginate (Fig. 4b). There was no significant difference in the percentage of total GAG retained in the tissues between the high and gradually increasing flow rate groups using PGA–alginate; the unreplicated measurement from the 5-day pre-culture experiment also gave a reasonably similar result (Fig. 8c). GAG lost into the medium from the PGA–alginate scaffolds accounted for 25–41% of total GAG in the cultures. These results show that adding alginate to the scaffolds reduced relative GAG losses compared with scaffolds without alginate even under high flow rate conditions; however, in contrast to the scaffolds without alginate, adjusting the medium flow rate or applying pre-culture treatment produced no additional benefit for the PGA–alginate cultures.

### Proteoglycan integrity

**Figure 9:**
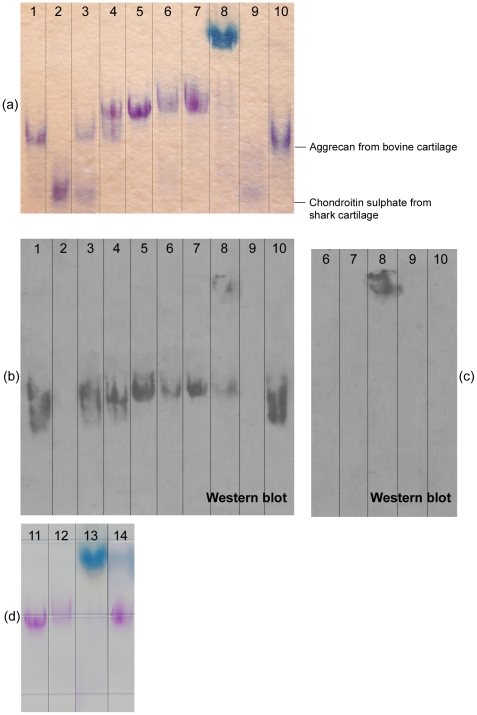
Analysis of proteoglycan size and integrity: (a and d) results from electrophoresis on composite acrylamide–agarose gels; (b and c) results from Western blots probed using monoclonal antibody specific to the hyaluronic-acid-binding region of human aggrecan. The back-up nitrocellulose membrane for capture of smaller-sized molecules is shown in (b); the primary membrane showing larger-sized molecules is shown in (c). Lane 1 – aggrecan from bovine cartilage; Lane 2 – chondroitin sulphate from shark cartilage; Lane 3 – a 1∶1 w/w mixture of bovine aggrecan and chondroitin sulphate; Lane 4 – a 1∶1 w/w mixture of bovine aggrecan and proteoglycans isolated from human fetal cartilage; Lane 5 – proteoglycans isolated from human fetal cartilage; Lanes 6 and 7 –proteoglycans isolated from tissue-engineered cartilage; Lane 8 – spent medium from bioreactor culture of tissue-engineered cartilage; Lane 9 – bovine aggrecan digested with papain; Lane 10 – aggrecan from bovine cartilage; Lane 11 – proteoglycans isolated from human fetal cartilage; Lanes 12, 13 and 14 – spent medium from bioreactor culture of tissue-engineered cartilage. The sample in Lane 12 was diluted and treated with 6 M urea; the sample in Lane 14 was treated with hyaluronidase.

Electrophoresis was used to investigate the size and integrity of aggrecan molecules in the cartilage constructs and bioreactor medium. As shown in Figure 9, all electrophoresed samples produced smears rather than sharp bands, reflecting the characteristic heterogeneity of proteoglycan composition and glycosylation [32]. The migration fronts of the bands are used to indicate the distance traveled by the samples. The three types of aggrecan tested, bovine aggrecan, aggrecan isolated from human fetal cartilage, and aggrecan isolated from tissue-engineered cartilage, were separated on the gel (Fig. 9a, Lanes 3, 4). Aggrecan from tissue-engineered cartilage (Fig. 9a, Lanes 6, 7) co-migrated with aggrecan isolated from human fetal cartilage (Fig. 9a, Lane 5): the small difference in aggrecan size most likely reflects slightly different post-translational modifications. All bands on the Western blots (Figs. 9b, 9c) reacted with antibody against anti-human aggrecan except chondroitin sulphate (Lane 2) and papain-digested bovine aggrecan (Lane 9).

Most untreated proteoglycan in the spent culture medium (Figs. 9a–9d, Lanes 8, 13) traveled only a very small distance on the gel. Medium samples produced a smear with a distinct blue color after toluidine blue staining (Fig. 9a), indicating the presence of proteoglycans and GAG, and stained strongly with anti-aggrecan antibody in the Western blot (Fig. 9c). Although some of the medium sample co-migrated with aggrecan isolated from tissue-engineered cartilage (Fig. 9b, Lanes 6–8), the large size of the proteoglycan complexes in the medium suggested the presence of aggregates that had not dissociated at the urea concentration used to prepare the samples. Further addition of urea to a diluted medium sample completely dissociated the aggregates (Fig. 9d, Lane 12); digesting the sample with hyaluronidase resulted in almost complete dissociation (Fig. 9d, Lane 14). With these treatments, medium samples produced aggrecan bands that traveled the same distance on the gel as aggrecan isolated from tissue-engineered cartilage (Fig. 9d, Lane 11).

These results demonstrate that the size of constituent proteoglycan molecules in the bioreactor medium was not significantly different from those within the cartilage constructs. In addition, medium proteoglycan was fully capable of aggregating and, consequently, incorporating into the developing cartilage matrix. As there was no evidence of proteoglycan degradation, loss of GAG from the constructs into the medium is attributed to simple diffusion or flushing out under the action of the perfusing medium rather than to fragmentation due to proteolytic cleavage or turnover.

## Discussion

Several strategies involving the manipulation of bioreactor hydrodynamics, pre-culture conditions, scaffold design, and seeding protocols were developed to reduce the loss of ECM components from cartilage constructs during bioreactor culture. For PGA scaffolds without alginate, applying a low or gradually increasing flow rate during bioreactor culture, or pre-culturing the scaffolds for 5 days prior to bioreactor culture, significantly improved the size and quality of the constructs compared with the non-perfused controls and bioreactor cultures operated at high flow rate without scaffold pre-culture (Figs. 1, 2, 3). The relative retention of GAG within the constructs was also improved markedly using these treatments (Figs. 4b, 4c). Together, these results suggest that moderated flow rates or scaffold pre-culture under benign hydrodynamic conditions protected early-formed ECM from being flushed away, allowing it to form a framework within the scaffold on to which other synthesized elements could accumulate before exposure to the full perfusion environment of the bioreactors. This finding is consistent with previous reports of reduced GAG accumulation in cultures perfused during the early stages of cartilage synthesis [10], [33] and with increasing medium flow rate [34], indicating that the beneficial effects of perfusion depend on first allowing deposition of some matrix around the cells as well as judicious control of the flow forces applied. In the current study, the relatively poor results using 2.5 weeks of pre-culture in T-flasks (Figs. 1a, 2a, 4) suggest that, whereas protection of the cells and developing matrix for several days before bioreactor culture was beneficial, 2.5 weeks was too long a period for the cells to maintain strong chondrogenic activity without the benefits of nutrient perfusion and hydrodynamic stimulation.

In contrast to the results with PGA scaffolds, the gradually increasing flow rate and 5-day pre-culture treatments had relatively little effect on construct quality and GAG retention in PGA–alginate scaffolds compared with cultures conducted at high medium flow rate (Figs. 5, 6, 7, 8). Yet, in many respects, the PGA–alginate scaffolds produced constructs with wet weights, biochemical composition, and GAG retention characteristics similar to or better than the maximum results obtained using PGA scaffolds without alginate (Figs. 5, 6, 7, 8 cf Figs. 1, 2, 3, 4). This suggests that the presence of alginate between the fibers of the scaffold protected the cells and developing matrix even at the highest flow rate tested and without scaffold pre-culture. The average pore size in alginate gel has been measured as 0.37±0.03 µm [35], which is much smaller than the pore dimensions of several hundred microns in fibrous PGA mesh [36]–[38]. As monomeric aggrecans extend to about 300 nm [39] and collagen fibers measure approximately 50 µm×240 nm [40], filling the interstices of PGA scaffolds with alginate can be expected to reduce strongly the release of these elements into the culture medium. This is consistent with overall specific rates of GAG release being an order-of-magnitude lower in the high flow rate cultures with PGA–alginate scaffolds compared with the high flow rate cultures and PGA alone (Figs. 4b, 8b).

Theoretically, the rate of transport of any component from the cartilage constructs into the medium depends on the porosity and other retentive properties of the scaffold and ECM, the magnitude of the shear forces acting on the construct, the surface area available for transfer, and the difference in component concentration between the tissue and medium. Consistent with the last factor in this list, the cumulative amounts of GAG released were generally higher in the better performing cultures that contained relatively high concentrations of GAG in the tissues (Figs. 2a, 4a, 6a, 8a). As well as GAG, collagen or procollagen may also have been released from the constructs. However, because hydroxyproline was present at relatively high concentration in the culture medium used, it was not possible to measure collagen release using analytical methods based on hydroxyproline. Concentrations of hydroxyproline in fresh culture medium and in samples of spent medium (*n* = 3) were found to be 360±11 µg mL^−1^ and 20±2.0 µg mL^−1^, respectively. It was thus difficult to distinguish between residual hydroxyproline provided in the medium and collagen or procollagen that may have been released from the developing tissues. The ELISA used for measurement of collagen type II was not applied to medium samples because of the high cost of analyzing the large number of samples generated by routine medium exchange. Although stripping of collagen from the PGA constructs remains a possibility, in contrast to the results found for GAG, there was no significant improvement in total collagen content compared with the non-perfused and high flow rate cultures when the low flow rate, gradually increasing flow rate, and 5-day pre-culture treatments were applied (Fig. 2b).

Construct shrinkage was observed using PGA scaffolds without alginate during the non-perfused control, high flow rate, and 2.5-week pre-culture experiments. Articular chondrocytes are known to express α-smooth muscle actin, a contractile actin isoform, and this has been related to the ability of chondrocytes to contract polymeric scaffolds during cartilage formation [41]. Scaffold contraction is generally undesirable because it alters the pore structure and shape of the scaffold; remedial strategies such as constraining scaffolds by clamping [42] or using highly cross-linked scaffold materials [43] have been employed. In the current work, contraction of PGA scaffolds under two of the bioreactor culture conditions tested resulted in some degree of medium by-passing with fluid flowing between the tissue and bioreactor wall. Nutrient and oxygen deprivation may have occurred in the constructs under these conditions, contributing to the poor tissue development and low GAG concentrations observed in the high flow rate and 2.5-week pre-culture experiments (Figs. 1a, 2a). The relatively high content of undissolved PGA fibers in the high flow rate constructs (Fig. 3g) is also consistent with medium by-passing.

Proteolysis of cartilage proteoglycan occurs continuously in the body throughout life; accelerated degradation of proteoglycans is a characteristic of diseases such as arthritis that damage the normal structure and function of cartilage [25]. The loss of GAG from cartilage constructs into the medium during bioreactor culture raises the question of whether these losses are due to simple flushing out of full-size molecules from immature and relatively porous tissues as a result of medium perfusion, or whether proteoglycans within the constructs are proteolytically degraded into smaller fragments, thus facilitating their removal. The integrity of proteoglycan aggrecan in the tissue-engineered constructs and medium was investigated using electrophoresis. Acrylamide–agarose gels were successful in separating very large and small proteoglycan and GAG molecules on the same gel without the need for sample purification or enzyme treatment of samples as required using SDS–PAGE [44], [45]. The results showed no evidence of proteoglycan degradation: after dissociation, aggrecan complexes in the bioreactor medium were similar in size to those in native human cartilage and within the tissue-engineered constructs (Fig. 9). Accordingly, loss of GAG from cultured tissues into the medium is attributed to simple removal of intact proteoglycan rather than to proteolytic cleavage or turnover.

Substantial improvements in GAG concentration, collagen type II concentration, and levels of collagen type II as a percentage of total collagen were obtained in this work by modifying the structure and composition of the scaffold and the conditions used for perfusion culture in bioreactors. The results demonstrate a direct link between cartilage construct quality and relative GAG retention. The first few days of culture were found to be critical for the proper formation of *de novo* tissue-engineered cartilage. Low flow rates are needed with porous scaffolds such as PGA only during the first week or so to protect early-deposited ECM until a macromolecular framework is developed to capture other synthesized elements. This was also achieved using a relatively short (5-day) pre-culture period before bioreactor operation. The presence of alginate gel within fibrous PGA scaffolds reduced the loss of ECM components from the constructs and obviated the need for flow rate modulation or scaffold pre-culture to protect the developing matrix.
